# Path analysis of regional logistics and economy coordinated development: An fsQCA approach

**DOI:** 10.1371/journal.pone.0297441

**Published:** 2024-02-01

**Authors:** Zhiyuan An, Enqiu He, Xue Du, Bo Sun

**Affiliations:** 1 College of Chemical Equipment, Shenyang University of Technology, Liaoyang, Liaoning, China; 2 Liaoning Provincial Institute of Grain Science, Liaoning Academy of Agricultural Sciences, Shenyang, Liaoning, China; Shandong University of Science and Technology, CHINA

## Abstract

The coordinated development of regional logistics and the economy is crucial for regional economic progress and for reducing regional development disparities. This study applies regional coordinated development theory and coupling theory, utilizing the Coupling Coordination Degree Model (CCDM) to analyze data from 31 provinces and cities in China in 2021, with the analysis results serving as the outcome variable. Additionally, we use data from four dimensions: infrastructure investment (II), technological innovation (TI), industrial structure (IS), and human capital (HC), as the conditional variables, conducting a multi-factor configurational analysis using fsQCA. Three paths with high coupling coordination and one path with non-high coupling coordination are identified, and the reasons for each path are analyzed. The results indicate that: 1) there are significant regional disparities in China regarding economic development, logistics development, and the degree of their coupling and coordination, with the eastern regions exhibiting higher levels and the western regions and other remote areas exhibiting lower levels. 2) The three paths with high coupling coordination are: “Infrastructure Investment—Technological Innovation”, “Technological Innovation—Industrial Structure—Human Capital”, and “Infrastructure Investment—Fundamental Innovation—Industrial Structure”. These three types facilitate the well-coordinated progress of regional logistics and the economy. The article concludes by highlighting policy suggestions that underscore the significance of fortifying the bond between the logistics industry and the economy, alongside earnest efforts to enhance regional logistics standards. This will foster a mutually reinforcing and co-developing situation, further promoting coordinated development among regions, achieving high-quality regional development, and reducing the imbalances in logistics and economic development among different regions.

## Introduction

Against the backdrop of rapid economic development in the past few decades, China’s development of industries has continuously expanded. According to data from the National Development and Reform Commission (NDRC), China’s total social logistics costs reached 17.8 trillion yuan in 2022, accounting for 14.7% of GDP. This not only indicates the rapid development of China’s logistics industry but also signifies the immense potential and room for growth in the Chinese logistics market. As a critical component of economic development, logistics plays a vital role in enhancing competitiveness, improving people’s quality of life, and promoting regional economic growth [1]. In other words, logistics has become a new momentum driving the fast development of national and regional economy.

However, the development of logistics further inevitably causes the disparity of regional economy among each regions, owing to reasons such as the restrictions of territory, the context of different regional development, the vast territory, and so on. [2]. Especially against the backdrop of rapid economic development in the past few decades, China’s industries and economy have continuously expanded. Take the western provinces of China as an example, it is far way from the coast, which exhibits a significant geographical contrast with the coastal provinces. This geographical difference results in notably higher transportation costs for the landlocked regions [3]. This effect is particularly pronounced in economically developed regions, which have accumulated long-term first-mover advantages [4]. However, this phenomenon inadvertently leads to economic setbacks in other regions and imparts a negative spillover effect on the economic growth of less-developed areas [5]. Thus, it is necessary to discuss the relationship between the development of regional logistics and the economy through a systematic study so as to clarify whether regional logistics develops harmoniously with its economy and analyze how to promote the coordinated relationship between both of them to further enhance the coordination between regional logistics and the economy so as to narrow the disparities between regions and promote high-quality sustainable development of the regional economy.

In recent years, the interaction between regional logistics and economic development has become an important research topic attracting wide attention from the academia [6–9]. For example, According to Du et al. [9], the development of regional economy and transportation has been a growing trend in the past decades, but the imbalance of coupling coordination degree in different regions in China is significant, namely, the eastern region is obviously higher than the central, western, and northeast regions. Lean et al. [10] use the dynamic structural model to test the relationship between logistics development and economic growth in both the short and long-run causality based on the data on Chinese logistics and economy. The finding implies economic development accompanying more demand for logistics and development. Xie et al. [11] apply the coupling coordination degree model(CCDM) to systematically study the coupling relationship between Chinese cold chain logistics and the economy from 2010 to 2019. Wang and Chen [12] measure the efficiency of the logistics industry in Wuhan city to evaluate the coordinated development of logistics development and low-carbon economy based on big data. Jiang et al. [13] employ the coupling coordination degree model and the spatial auto-correlation analysis model by constructing the evaluation index system of the logistics industry and regional economy in the YRDR(Yangtze River Delta Region) to reveal the different regional coordinated levels. Shu et al. [14] take system theory and coupling theory as the analytical framework to elaborate the relationship between the digital economy and rural logistics.

These studies provide valuable insights into regional logistics and the economy through diverse strategies and measures, they examine further the relationship between logistics and the economy. However, those current works seldom explore how to promote the coordination level between regional logistics and the economy in diverse regions [15, 16], most prior studies concerning the mutual relationship and coordinated relationship between regional logistics and the economy have predominantly centered on qualitative analysis and factorial analysis.

Thus, there needs to be more systematic research to analyze and fill the significant research gap that currently exists. This paper employs empirical methods based on the coupling theory and coordinated development theory, the Coupling Coordination Degree Model (CCDM) of quantitative analysis is adopted in the paper to investigate the coupling coordinated development context between regional logistics and the economy. Additionally, in comparison to existing research, this study adopts a configurational analysis perspective to clarify multiple factors and their combinations that affect the coordinated development of regional logistics and economy. In contrast to conventional regression analysis techniques, configuration methods can make configurational classifications and judgments based on the simultaneous consideration of multiple interdependent factors [17]. In other words, configuration methods possess the capacity to deliberate upon multiple potential factors that give rise to substantial disparities in their collective influence on the outcome. These factors may either complement or substitute for one another, resulting in significant variations in their combined impact on the coordinated development of regional logistics and the economy. While traditional regression analysis methods can pinpoint the roles played by various factors in the relationship between regional logistics and the economy, they often treat each factor in isolation, potentially neglecting the interplay among key variables.

This study will analyze the complementarities and synergistic mechanisms using fsQCA to facilitate the achievement of synergistic effects in regional logistics and the economy. A systematic analytical framework will be developed to comprehensively assess the impacts of. The framework will consider multiple factors, including infrastructure investment (II), technological innovation (TI), industrial structure (IS), and human capital (HC). It will assess the potential for the coordinated development between regional logistics and the economy across different regions using quantitative and qualitative methods.

Based on the above analysis, this article aims to address the following three research questions:

- RQ1. How can the level of coupling and coordination between regional logistics and the economy be reflected?
- RQ2. Which factors can influence the level of coupling and coordination between regional logistics and the economy, and what evidence can be used to determine them?
- RQ3. How are the paths to synchronized progress of logistics and the economy different in various regions?

The subsequent sections of this paper are structured as follows: The Literature review and research framework section introduces the literature review of regional logistics and economy, the factors affecting the coordinated development between regional logistics and the economy, and the framework established by related works; The Materials and methods section first introduces the methods used, namely the entropy weight method (EMM), Coupling Coordination Degree Model (CCDM) and fsQCA. Then, introducing the data collection method and source, determining variables, and data description are carried out. The Empirical analysis section conducts thorough empirical analysis, analyzing the different results and paths of the configuration. The remaining sections of the paper provide a summary analysis of the entire paper, propose policy recommendations, and elaborate on the limitations of the research and future research directions.

## Literature review and research framework

### Literature review

Logistics, recognized as a pivotal driver of economic development, has emerged as a dynamic force propelling the rapid growth of both national and regional economies [18]. Notably, the development of logistics infrastructure, encompassing transportation, warehousing, and port logistics, serves as a pivotal benchmark for evaluating logistics advancement. Rational investments in infrastructure can stimulate heightened demand for goods and services, a phenomenon particularly conspicuous in underdeveloped regions [7]. These investments yield reductions in transit time and associated costs. Meanwhile, robust regional economic performance forms a steadfast economic cornerstone, coupled with reliable technological underpinnings. This proposition is corroborated by Zhang [19], who posited that enhancements to transportation infrastructure can propel economic growth and foster equilibrium in China’s diverse economies. This effect is particularly prominent in the central and western regions, where transportation infrastructure lags. Augmented investments in this sector can galvanize rapid regional economic expansion.

In turn, regional logistics fosters opportunities for regional economic development [10, 20]. Lean et al. [10] empirically assessed the relationship between logistics and economic growth in both the short and long term, revealing that economic progress spurs heightened demand for logistics services. From a cost perspective, Zhang [19] scrutinized regional economic growth and inter-regional transportation across 29 Chinese provinces, employing the Cobb-Douglas production function framework. The outcomes unveil a positive correlation between the gradual reduction of inter-regional transportation costs and the diminishment of economic disparities, thereby expediting the harmonized development of distinct regions. Furthermore, Zhang [7] delved into the nexus between logistics development and economic growth within diverse Chinese regions. This inquiry employed an output model and Hausman Test on panel data spanning from 1997 to 2016, encompassing four hierarchical regional levels in China. The results highlight the substantive influence of regional logistics development on the economic growth of each respective region. Remarkably, all evaluated indicators manifest a favorable correlation with economic growth across the diverse regions.

However, challenges persist in the development of logistics and regional economies. As early as 2002, Jiang and Prater [21] outlined significant issues in China’s logistics distribution, including underdeveloped infrastructure, governmental regulations, regional protectionism, and fragmented distribution networks spanning the entire nation. Over two decades later, substantial progress has been achieved in mitigating these issues. Nevertheless, the scope and nature of these challenges continue to diverge notably among regions characterized by varying levels of development and geographical attributes. Specifically, China’s earlier open-door policy, initiated by the government in 1978, led to substantial foreign investment in coastal provinces. This policy accentuated geographical disparities among China’s regions. Western provinces, being landlocked with considerable distances to the coast, exhibit a significant geographical contrast with their coastal counterparts in the east. This geographical difference results in notably higher transportation costs for the landlocked regions [3]. Furthermore, the integration of economic activities across diverse regions through logistics operations serves to reduce transportation expenses for businesses and residents, while enhancing the accessibility and appeal of specific regions. This effect is particularly pronounced in economically developed regions, which have accumulated long-term first-mover advantages [4]. However, this phenomenon inadvertently leads to economic setbacks in other regions and imparts a negative spillover effect on the economic growth of less-developed areas [5]. Consequently, there is an imperative need to further enhance the coordination between regional logistics and the economy.

Synchronized progress among regions constitutes a pivotal element of regional integration [22]. As research on regional integration continues to deepen, it is increasingly recognized as an effective strategy for harnessing development advantages and promoting overall progress [23, 24]. Logistics helps to reduce regional disparities, increase the demand for goods and services, reduce transit time, and lower associated costs, which contribute to the regional economy in a direct or indirect way [3, 18, 25]. At the same time, a strong regional economic level can provide a solid economic foundation and reliable technology, which in turn can stimulate the development of regional logistics [14, 26]. Within this context, an expanding body of scholars has placed greater emphasis on the synergistic interplay between regional logistics and the economy and the roles they assume. Lan [6] employed Bayesian Network analysis to explore the reciprocal dynamics of coordinated development between metropolitan economies and logistics. The results underscore the pivotal role of expediting the fundamental construction of urban logistics infrastructure as a prerequisite for sustaining the coordinated development of the economy and logistics. Furthermore, it was argued that the level of development significantly influences the coordinated development of the economy and logistics. Yang et al. [26] conducted a quantitative study employing big data and the Haken model to demonstrate the essential role of economic development in the synchronized advancement of metropolitan logistics and the economy. Du et al. [9] employed quantitative methodologies to examine the state of coordination between regional economies and transportation. They underscored the critical role of advancing coordination as a foundation for fostering regional sustainable development. Thus, the mutually beneficial and developmental relationship between regional logistics and the economy, where they interact and promote each other.

In summary, the results of investigations and studies make important and unique contributions to the coordinated progress in diverse strategies and perspectives. They indicate that there is a mutual relationship between logistics development and economic growth. In other words, the development of logistics promotes economic growth, and economic growth also stimulates logistics development. For one thing, regional coordination and development can address the imbalances in regional economic development [9]. For another thing, it also can contribute to improving the social, industrial, and demographic structures within the regions. Furthermore, regional coordinated and sustainable development are beneficial for stimulating economic growth within the regions [16].

#### Infrastructure investment

Infrastructure plays a crucial role in the development of regions and nations [27]. According to the neoclassical economic theory [28], development proceeds as firms and households make more efficient use of labor, capital, and natural resources. Infrastructure therefore has positive effects on regional growth. For one thing, its availability should increase the productivity of physical and human capital, if this results in lower production and logistics costs, demand for the output of the region should increase. Nevertheless, the level and quality of infrastructure directly influence the productivity of businesses and regional economic growth [29], and different investments in infrastructure capital lead to regional and national inequalities [30]. By breaking the regional segregation, infrastructure can enable small and closed markets to connect and achieve a significant market size. For example, Hong et al. [31] pointed out that geographical advantages and government policies favoring coastal areas have led to significant disparities in infrastructure, resulting in substantial differences between regions. Li et al. [25] shared this view and highlighted the impact of logistics infrastructure on regions. They suggested that appropriate infrastructure investments can help narrow the gap between inland and coastal areas and promote economic growth.

For another thing, if infrastructure also serves as a direct factor input, then higher levels of investment should raise regional output [32]. In the other words, infrastructure can also enhance regional collaboration and spillovers. Lan and Zhong [6] emphasized the critical role of various factors in the coordinated development between logistics and the economy, with logistics infrastructure being a prerequisite for accelerating coordinated development. Qi et al. [33] examined the possibility of spatial spillover effects of logistics infrastructure in Chinese regions, and concluded that the suitable infrastructure investment in states with different development levels could promote regional economic development.

**Proposition 1**: Infrastructure investment is an enabler for the coordinated development between regional logistics and the economy

#### Technological innovation

In alignment with the principles of regional development theory [34], technological innovation assumes a central role in steering the synchronized progress of regional logistics and the economy in the contemporary era. Particularly within the context of China’s economic transformation, where the demographic dividend is gradually waning and environmental concerns are assuming greater significance, achieving a successful transition in economic development necessitates a shift away from traditional development models towards innovation-driven growth, as opposed to relying solely on factor-driven expansion [35].

From a macroscopic standpoint regarding the region’s economic advancement, technological innovation facilitates the highly efficient conversion of technological advancements. By enhancing the effectiveness of enterprise innovation, it not only generates increased business value for the technology or product but also opens new market opportunities, ultimately leading to economic growth. The clustering of regional industries through knowledge sharing and the diffusion of technology results in the creation of a regional innovation network, thereby promoting industrial upgrades and establishing new focal points for economic growth [36].

In the realm of the logistics industry, emerging technologies, driven by technological innovation, such as logistics networks, big data, and automated logistics equipment, are experiencing rapid expansion. These breakthroughs are initiating profound transformations within the global logistics sector. This rapid technological progress is a direct response to the substantial challenges faced by the logistics industry. These challenges include complex monitoring processes, management inefficiencies, container shortages, price fluctuations, unpredictable transport planning, and other factors that introduce uncertainty into the industry’s development. Furthermore, issues such as obscured information and communication obstacles resulting from data silos, as well as the supply chain’s “bullwhip effect,” represent critical pain points within the industry [37, 38]. In summary, technological innovation has the potential to reduce resource consumption and logistics costs, thereby enhancing the quality of logistics services, promoting the transformation and modernization of industrial structures, and facilitating regional economic progress.

**Proposition 2**: Technological innovation is a necessary condition for the coordinated development between regional logistics and the economy.

#### Industrial structure

Regional industrial structure may help to explain differences in economic performance and dynamics between regions with a similar level of industrial agglomeration [39]. For one thing, regional industrial structure is of great importance to firm productivity and economic growth potential [40]. A diverse industrial structure would also enhance productivity and promote innovation through cross-fertilization, have a positive impact on value-added growth, and be very important for the development of high-tech industry and the attraction of new firms. The larger the regional market capacity is, the more favorable it is to attract enterprises to gather in the region [41]. The regional industrial structure upgrade had a spatial spillover effect, and regions with strong industrial structure upgrade capabilities had a positive impact on the surrounding areas. In the other words, industrial structure upgrading is an inevitable trend of economic development and has become an important driving force for economic growth [41, 42], the key to achieving sustainable economic growth lies in structural transformation and industrial upgrading, however, there is regional heterogeneity in the upgrading of industrial structure, mainly due to the imbalance of regional economy [43]. In the other words, there is an influence of the regional industrial structure on the regional economic progress.

For another thing, as a derivative demand of regional economic development [44], the industrial structure can affect the evaluation of the regional logistics and promotes the comprehensive competitiveness of the logistics [41]. For example, Shevchenko et al. [45] conducted a historical analysis and integrated it with the new structural economics theory, revealing a mutual promotion effect between the upgrading of the intelligent logistics industry and industrial structure. Liu et al. [46] constructed a VECM model using Jiangxi Province as an example to analyze the effects of industrial structure adjustment on the development of the regional logistics industry in the perspectives of long-term and short-term. The findings revealed that the contribution rate of the first the second industries to the development level of logistics in Jiangxi province is larger, and the variance contribution rate of the third industry is small, which proves that the change and adjustment industrial structure have a impact on the development of the regional logistics industry in region.

**Proposition 3**: Industrial structure is an enabler for the coordinated development between regional logistics and the economy.

#### Human capital

Modern economic development theories underscore the pivotal role of labor migration in facilitating skill enhancement through human capital spillovers from high-skilled individuals to neighboring lower-skilled workers [47]. To put it differently, human capital, serving as a metric for human resources, can serve as a catalyst for regional economic development [48]. This is because the majority of social advantages stem from the accumulation of human capital, which exerts a profound influence on long-term economic progress [49].

According to the economic growth and spatial equilibrium theories [50], which accentuate the rising returns associated with human capital and agglomeration, the evaluation of regional economic development within a given area becomes feasible. The concentration of human capital in a particular region offers incentives for labor migration, thereby reinforcing disparities in human capital concentration and economic development [51]. Specifically, while human capital remains indispensable for sustaining long-term regional growth in the logistics industry, there exists a substantial disparity between the educational skills demanded by the logistics sector. This incongruity poses a genuine threat to economic growth and hampers the competitive edge of the logistics industry [52]. Addressing this issue is imperative, as the geographical and economic disparities in China are poised to exacerbate the existing imbalance.

**Proposition 4**: Human capital is a necessary condition for the coordinated development between regional logistics and the economy.

In summary, the synchronized progress of regional logistics and the economy is subject to a myriad of influencing factors and reasons, with the four dimensions mentioned earlier potentially being among these influential factors. We adopt a perspective that places particular emphasis on different facets affecting the coordinated development between regional logistics and the economy in our exploration of these influencing factors. Drawing on theoretical foundations from the existing literature, this paper constructs its conceptual framework for the coordinated development of regional logistics and the economy, leveraging the configurational effect. In general, for an analysis of the coordinated relationship between regional logistics and the economy, it is imperative to consider the contributions of each factor, such as economic development, technological advancements, governmental policies, and other relevant indicators. Furthermore, the interactions among these factors play a crucial role, an aspect that has been regrettably overlooked in the existing body of literature.

We address the gaps in the existing literature and contribute to it in the following dimensions. Firstly, despite the apparent maturity of this field of research, prior studies concerning the coordinated relationship between regional logistics and the economy have predominantly centered on qualitative analysis and factorial analysis. Only a fraction of the research has delved into strategies for promoting coordinated development between the two (e.g. [16]). Therefore, it is imperative to utilize the coupling theory model to explore the intricate interaction mechanisms between the subsystems of regional logistics and the economy. This approach allows us to unveil the degree of coordination between them and provides an effective means of conducting path analysis. Secondly, previous research has tended to overlook potential interactions among key factors. However, it is worth noting that these factors may either complement or substitute for one another, resulting in significant variations in their combined impact on the coordinated development of regional logistics and the economy. Thirdly, the research methods employed in previous studies warrant scrutiny. While traditional regression analysis methods can pinpoint the roles played by various factors in the relationship between regional logistics and the economy, they often treat each factor in isolation, potentially neglecting the interplay among key variables. Lastly, earlier studies failed to identify critical pathways influenced by variations. While scholars have confirmed the existence of influential relationships among various factors and the level of coordinated development between regional logistics and the economy, they have been unable to establish the complementary associations that exist among these factors.

To address the lack of a holistic understanding of the nexus between regional logistics and the economic research on regional economic development, we undertake a coupling coordination model, CCDM, to identify the development level of the logistics industry and economy by establishing the evaluation index system and the way to calculate the synergy degree between both of them first. The coordination between regional logistics and the economy includes some subsystems such as politics, economy, infrastructure construction, and so on. Its coordinated development depends not only on the construction level of each subsystem but also on the degree of coordination between all the systems. Coupling theory reflects the degree of interaction and coordination between systems through coupling degree and coupling degree, so as to quantitatively judge whether systems develop harmoniously. The coupling theory is an effective method to test the relationship between two or more interacting systems. However, most coupling theory research focuses on the coupling between regional logistics and the economy and less on the coupling relationship between regional logistics and the economy.

Meanwhile, the key factors affecting the coordination between regional logistics and the economy were identified. Then we adopt the fuzzy-set qualitative comparative analysis (fsQCA) technique, which allows for “configurational classifications and judgments based on the simultaneous consideration of multiple interdependent factors” [17]. We analyze the path analysis of the coordination between regional logistics and the economy through fsQCA. Based on the analysis results, our study provides decision-making support and an analysis approach for achieving the goal of the promotion of coordinated development between regional logistics and the economy in 31 provinces of China.

After the above discussion, the research framework for this study has been established and is clearly illustrated in S1 Fig.

## Materials and methods

### Entropy weight method (EMM)

Entropy was first introduced to information theory by Shannon [53]. Currently, it has been widely applied in various fields, such as engineering and social economics. The basic idea of entropy weighting method (EWM) is to determine the objective weight based on the variability of indicators. Generally speaking, if the entropy *E*_*j*_ of an indicator is smaller, it indicates that the degree of variability of the indicator value is greater and provides more information. Therefore, it can play a greater role in comprehensive evaluation and its weight is also larger. Conversely, if the entropy *E*_*j*_ of an indicator is larger, it indicates that the degree of variability of the indicator value is smaller and provides less information. Therefore, it can play a smaller role in comprehensive evaluation and its weight is also smaller.

1. Standardized processing of original data
  $$\begin{array}{c} Y_{ij}=\frac{X_{ij}-\text{min}\;(X_{j})}{\text{max}\;(X_{j})-\text{min}\;(X_{j})} \end{array}$$ (1)
  $$\begin{array}{c} Y_{ij}=\frac{\text{max}\;(X_{j})-X_{ij}}{\text{max}\;(X_{j})-\text{min}\;(X_{j})} \end{array}$$ (2)
2. Calculate the entropy of each indicator
  According to the definition of information entropy in information theory, the formula for the entropy of a set of data is:
  $$\begin{array}{c} E_{j}=-\text{ln}{\left(n\right)}^{-1}\sum_{i=1}^{n}p_{ij}\times \text{ln}\;p_{ij} \end{array}$$ (3)
  The *p*_*ij*_ value is calculated as follows:
  $$\begin{array}{c} p_{ij}=\frac{Y_{ij}}{\sum_{i=1}^{n}Y_{ij}} \end{array}$$ (4)
3. Determine the weightings of each indicator
  The entropy weight of each index is calculated by Eq 5, where k represents the number of indicators. The results are shown in Table 1.
  $$\begin{array}{c} W_{j}=\frac{1-E_{j}}{k-\sum E_{j}} \end{array}$$ (5)

**Table 1:** Indicators’ weighting for regional logistics and regional economic.

Dimensions	Indicators	Weights	References
**Indicators for regional logistics**	A total length of postal routes	0.223	Hooi et al. [10], Cheng and Peng [55], Lan and Tseng [54], etc.
	Highway freight vehicle quantity	0.109	
	Freight volume	0.076	
	Cargo turnover	0.144	
	Number of postal service outlets	0.090	
	Total postal business volume	0.233	
	Number of railway transportation practitioners	0.053	
	Number of people in transportation, warehousing, and postal industries	0.072	
**Indicators for regional economic**	Regional GDP	0.092	Lan and Tseng [54], Huang [57], Chen and Zhang [42], etc.
	Total retail sales of consumer goods	0.101	
	Total import and export volume	0.218	
	Added value of primary industry	0.077	
	Added value of secondary industry	0.106	
	Added value of tertiary industry	0.099	
	Per capita regional GDP	0.093	
	Local fiscal revenue	0.099	
	Per capita disposable income	0.115	

### Coupling coordinated degree model (CCDM)

The CCDM model is used to measure the coordination relationship between multiple systems. It is a mature model and has been widely used in many fields, such as the coordinated development between regional logistics and economy, the coordinated degree between ecological environment, and so on [9, 54]. The following are the detailed steps to construct it:

1. Constructing Indicator System First, determinating logistics indicators. Determining logistics indicators is challenging because there is no specific standard for measuring logistics indicators so far. Currently, many scholars consider transportation, human capital, postal services, communication, and other aspects as the determinants of logistics indicators. Hooi et al. [10] demonstrated through empirical analysis that transportation network infrastructure plays a crucial role in China’s logistics system and elaborated on the significant differences in logistics levels between different regions within China. Cheng and Peng [55] analyzed the relationship between logistics industry and economy in Anhui Province by taking the volume of goods turnover as the research object. Gan et al. [56] conducted a comprehensive analysis of the logistics system, considering logistics infrastructure, logistics development scale, industrial structure, and profit level as key aspects. Lan and Tseng [54] determined the indicators that reflect logistics capabilities as the total fixed asset investment, the number of operating trucks, the number of logistics employees, the total length of postal routes, the operating revenue of road freight, the total volume of postal services, and the total mileage of highways.
  Second, identifying economic indicators. The composition of economic indicators has been informed by the research of numerous scholars in the field. According to Huang [57], per capita Gross Domestic Product (GDP) is a measure of the standard of living of a country’s population and is defined as the ratio of the total GDP to the resident population of a region. It is one of the most important macroeconomic indicators and plays a crucial role in measuring economic strength and development. Lan and Tseng [54] determined economic evaluation indicators as various indicators that can reflect regional economic development, including the added value of the tertiary industry, per capita GDP, total retail sales of social consumer goods, total fixed asset investment, per capita disposable income, household consumption level, and public fiscal revenue. Chen and Zhang [42] defined the indicators of regional economic development level as the level of population consumption, GDP growth rate, and retail sales of social consumer goods.
  In summary, in this paper, regional economic development indicators were determined, including 8 logistics development indicators: total length of postal routes, quantity of road freight trains, freight volume, cargo turnover, number of postal business outlets, total postal business volume, number of railway transport industry employees, and number of transportation, warehousing, and postal service employees. In addition, 9 regional economic development indicators were included: regional GDP, total social retail sales of consumer goods, total import and export volume, added value of the primary/secondary/tertiary industry, per capita regional GDP, local fiscal revenue, and per capita disposable income. The indicator system and the weight of each indicator are shown in Table 1.
2. Calculating the Degree of Coupling Development
  The coordination between regional logistics and the economy includes some subsystems such as politics, economy, infrastructure construction, and so on. Its coordinated development depends not only on the construction level of each subsystem but also on the degree of coordination between all the systems. Coupling theory reflects the degree of interaction and coordination between systems through coupling degree and coupling degree, so as to quantitatively judge whether systems develop harmoniously. The coupling theory is an effective method to test the relationship between two or more interacting systems. The CCD model is used to measure the coordination relationship between multiple systems. It is a mature model and has been widely used in many fields, such as the coordinated development between regional logistics and economy, the coordinated degree between ecological environment, and so on [9, 54]. However, most coupling theory research focuses on the coupling between regional logistics and the economy and less on the coupling relationship between regional logistics and the economy.
  The Coupling Coordination Degree (CCD) is a method used to measure the consistency and interaction between elements within or between systems. The conventional formula for measuring the degree of coupling is:
  $$\begin{array}{c} C={\left[\frac{\prod_{i=1}^{n}U_{i}}{{\left(\frac{1}{n}\sum_{i=1}^{n}U_{i}\right)}^{n}}\right]}^{\frac{1}{n}} \end{array}$$ (6)
  In the equation, *n* represents the number of subsystems, *U*_*i*_ is the standardized value of the i-th subsystem, and *α*_*i*_ is the weight of the i-th subsystem. The coupling degree C ranges from 0 to 1, with lower values indicating greater disparity between the subsystems and higher values indicating greater coupling. Although the coupling degree can reflect the strength of interaction, it cannot reflect the level of coordinated development between the systems. Therefore, it is necessary to introduce a coupling coordination model, as shown in Eq 7.
  $$\begin{array}{c} T=\sum_{i=1}^{n}\alpha_{i}\times U_{i} \end{array}$$ (7)
  The coupling degree model is capable of assessing the correlation between subsystems; however, it has certain limitations. For instance, it may yield high values for the coupling degree even when the subsystem’s development level is low. To overcome this drawback, a coupling coordination model is employed to gauge the degree of synchronized development between two subsystems, ensuring a more comprehensive evaluation [58], as shown in Eq 8.
  $$\begin{array}{c} D={\left(C\times T\right)}^{\frac{1}{2}} \end{array}$$ (8)
  The classification of regional logistics and economic coupling coordination levels is shown in Table 2.

**Table 2:** Classification criteria for coupling coordination degree level.

D-Value Interval	Level	Degree	D-Value Interval	Level	Degree
(0.0~0.1)	1	Extreme disorder	[0.5~0.6)	6	Barely coordination
[0.1~0.2)	2	Severe disorder	[0.6~0.7)	7	Primary coordination
[0.2~0.3)	3	Moderate disorder	[0.7~0.8)	8	Intermediate coordination
[0.3~0.4)	4	Mild disorder	[0.8~0.9)	9	Good coordination
[0.4~0.5)	5	Near coordination	[0.9~1.0)	10	High quality coordination

### fsQCA

Fuzzy Set Qualitative Comparative Analysis (fsQCA), developed by sociologist Charles Ragin, is a set-theoretic causal analysis method that provides a means of analyzing causal relationships between conditions and outcomes [59]. Unlike other correlation-based quantitative analytical methods, such as traditional regression analysis, fsQCA attempts to establish logical relationships between causal conditions and outcome combinations, enabling the study of complex, interactive relationships between multiple variables in diverse environments, where different combinations of causation ultimately lead to the same expected outcome [60]. Moreover, fsQCA is capable of analyzing small-n data [61], which enabled this study to analyze a sample of 31 provinces and cities. This study uses fsQCA4.0 software [62].

### Data collection

The data sources are “China Statistical Yearbook,” data released on the website of the National Bureau of Statistics of China (http://www.stats.gov.cn/), and local statistical yearbooks of 31 provinces and cities.

### Variables definition

#### Outcome variable

The result variable represents the coupling coordination of regional logistics and economy in 31 Chinese provinces and cities (excluding Taiwan, Hong Kong, and Macao), as shown in Table 3. The results indicate a significant disparity in the synchronized progress of regional logistics and economy in China. The eastern region, including the Yangtze River Delta region, Pearl River Delta region, and other areas, exhibits a high degree of coupling, while there is a low coupling in the central and western regions, especially in Tibet, Qinghai, Gansu, Ningxia, and other regions.

**Table 3:** Logistics and economic coupling coordination degree.

Region	D-Value Interval	Coordination Level	Region	D-Value Interval	Coordination Level
Guangdong	0.985	10	Guangxi	0.483	5
Shandong	0.878	9	Shaanxi	0.477	5
Jiangsu	0.876	9	Chongqing	0.469	5
Zhejiang	0.759	8	Yunnan	0.452	5
Henan	0.703	8	Inner Mongolia	0.445	5
Shanghai	0.694	7	Heilongjiang	0.42	5
Hebei	0.684	7	Xinjiang	0.394	4
Anhui	0.629	7	Tianjin	0.375	4
Sichuan	0.622	7	Jilin	0.353	4
Beijing	0.58	6	Guizhou	0.334	4
Hubei	0.576	6	Gansu	0.272	3
Fujian	0.568	6	Hainan	0.24	3
Liaoning	0.543	6	Ningxia	0.225	3
Hunan	0.539	6	Qinghai	0.151	2
Shanxi	0.497	5	Tibet	0.1	2
Jiangxi	0.497	5			

Notes: the above data is arranged in descending order based on the coupling coordination degree D value.

#### Conditional variable

This paper combines the research findings of many scholars and uses infrastructure investment, technological innovation, industrial structure, and human capital as the conditional variables to conduct a multifactor configuration analysis. The conditional variables and their data source references are shown in Table 4.

**Table 4:** Logistics and economic coupling coordination degree.

Dimensions	Indicators	Weights	References
**Infrastructure Investment (II)**	Cargo volume	0.160	Lan and Zhong [6], Wang and Deng [63], Yang and Li [64], etc.
	Transportation network	0.468	
	Added value of logistics industry	0.129	
	Transportation expenditure	0.179	
	Growth value of fixed asset investment in infrastructure	0.063	
**Science and R & D (TI1)**	Science and technology practitioners	0.116	Freeman and Soete [65], Flor and Oltra [66], etc.
	Scientific and technological expenditure	0.154	
	Industrial enterprise R&D expenses	0.174	
	The sales revenue of new products in enterprises	0.189	
	Domestic patent application acceptance volume	0.177	
	Technology market turnover	0.190	
**Information Technology (TI2)**	Information industry employees	0.418	
	Investment growth value in information technology services	0.037	
	E-commerce sales revenue	0.385	
	Internet subscribers	0.160	
**Industrial Structure (IS)**	Value added of primary industry	0.274	Qu et al. [67], Shevchenko et al. [45], etc.
	Value added of secondary industry	0.376	
	Value added of tertiary industry	0.351	
**Human capital (HC)**	Number of higher education students	0.291	Mazelis [68], Chi [69], etc.
	Investment in education	0.405	
	Number of logistics professionals	0.304	

Infrastructure Investment (II): Nine indicators including freight volume, transportation network, and added value of the logistics industry are selected as evaluation standards. Technological Innovation (TI): Divided into two categories of Science and R&D (TI1) and IT Industry (TI2), indicators such as patent quantity, technology expenditure, and information industry investment growth value are selected. Industry Structure (IS): The proportion of the second and third industries in GDP is selected as the indicator. Human Capital (HC): Divided into two aspects of education investment and employment, indicators such as the number of university students, education funding, and the number of employees in the logistics industry are selected.

### Variable calibration

The first step in analyzing fsQCA is data calibration. Calibration is a necessary and routine operation in fields such as chemistry, astronomy, physics, and other natural sciences, where researchers calibrate their measuring devices to established standards [59]. There are two main methods for data calibration in QCA analysis: direct calibration method and indirect calibration method. The direct calibration method calibrates variables using three anchor points that are directly determined. The indirect calibration method determines the calibration through the actual distribution of the sample and theoretical knowledge. When there are no standards or substantive research externally, calibration can be based on sample percentiles [70]. Regardless of the method used, calibrated values range between 0.0 and 1.0 [59]. Following Ragin’s [59] recommendation, this study employs a direct calibration method, which uses three anchors to structure a fuzzy set. The anchors include the thresholds for full membership (5%), full non-membership(95%), and non-membership(50%). The original data was converted to membership using the calibrate (x, n1, n2, n3) function in fsQCA4.0 software [62]. The variable calibration results and descriptive statistics for each variable are shown in Table 5.

**Table 5:** Variable calibration results and descriptive statistical analysis.

Variable	Calibration Results			Descriptive statistical					
	Full memership	Crossover point	Full non-membership	Variable	Mean	Median	Standard deviation	Min	Max
**II**	0.335	0.070	0.014	II	0.122	0.070	0.181	0.000	1.000
**TI1**	0.797	0.102	0.004	TI1	0.192	0.102	0.251	0.000	1.000
**TI2**	0.770	0.121	0.010	TI2	0.199	0.121	0.242	0.000	1.000
**IS**	0.798	0.211	0.015	IS	0.281	0.211	0.242	0.000	1.000
**HC**	0.579	0.228	0.005	HC	0.255	0.228	0.208	0.000	1.000
**CCD**	0.877	0.497	0.188	CCD	0.510	0.497	0.206	0.100	0.985

## Empirical analysis

In the present investigation, we employed fsQCA 4.0 and SPSS software to derive our findings. Subsequent to the calibration process, we scrutinized the necessity conditions associated with four distinct condition variables, namely infrastructure investment (II), technological innovation (TI), industrial structure (IS), and human capital (HC). Following this, we conducted a thorough sufficiency analysis from a configuration-centric standpoint, along with a rigorous examination of the robustness of our results.

### Necessity analysis

After calibrating fuzzy set variables, conducting necessary condition tests for each variable is imperative [71]. Furthermore, importing sample data fuzzy membership scores into the fsQCA4.0 software is essential. In this study, necessary conditions for the outcome variable were identified as antecedent conditions with a consistency level greater than 0.9 and a coverage level exceeding 0.5 [72]. Conversely, antecedent conditions with lower consistency are regarded as insufficient for constituting necessary conditions. As demonstrated in Table 6, IS, ~TI1, ~TI2, ~IS, ~HC exhibit consistencies above 0.9 and coverage levels above 0.5, they necessitate further analysis as they are likely to be direct causes of coordinated development levels between regional logistics and the economy. In accordance with Schneider’s authoritative work (2012), if a conditional variable passes the consistency test with a threshold above 0.9, it is considered a necessary condition. In essence, the conditional variable encompasses the outcome variable entirely, thus covering all cases of inclusion in the outcome variable. To determine further necessity, it is recommended to employ an XY plot, as depicted in S2 Fig. Trivialness of a necessary condition can arise from two sources: when the magnitude of X exceeds that of Y, or when X and Y approach constants. Both conditions must be thoroughly considered, and in either of these situations, no condition should be interpreted as necessary. [73]. Furthermore, Zhang [74] and Tan et al. [75] highlight the inability of a conditional variable to serve as a necessary condition for the outcome variable if approximately one-third of the data points lie above the diagonal line in the XY plot. It can be concluded that the conditions “~TI1,” “~TI2”, “~IS”, and “~HC” play pivotal roles in contributing to the non-high coupling scenario. This suggests that technological innovation and human capital are the primary factors responsible for the absence of high coupling and coordinated development between regional logistics and the economy. Other antecedent conditional variables have limited explanatory power for coordinated development extent between regional logistics and the economy, as their consistencies are all below 0.9. Accordingly, we scrutinized these causal conditions in the truth table analysis of fsQCA to investigate the various control mechanisms that could lead to coordinated development between regional logistics and the economy.

**Table 6:** Analysis of necessary conditions.

Variable	High coupling degree		Non-high coupling degree	
	Consistency	Coverage	Consistency	Coverage
**II**	0.808	0.889	0.506	0.560
**~II**	0.600	0.547	0.899	0.825
**TI1**	0.801	0.952	0.406	0.485
**~TI1**	0.566	0.486	0.960	0.829
**TI2**	0.798	0.933	0.429	0.505
**~TI2**	0.576	0.501	0.943	0.824
**IS**	0.905	0.959	0.473	0.504
**~IS**	0.532	0.501	0.961	0.911
**HC**	0.863	0.921	0.476	0.511
**~HC**	0.542	0.507	0.926	0.872

Notes: The symbol (~) indicates the negation of the attribute.

### Constructing the truth table

After testing for necessity, it is necessary to set thresholds for sufficient conditions. The sufficient condition indicates that specific causal conditions, whether individually or in combination, are able to effectively lead to the outcome. Once the calibration process is completed in the fsQCA4.0 program, a truth table will be constructed [76]. The truth table is a key tool that reflects the complexity of causal relationships within a system [59]. The frequency threshold is set at 1.5% of the sample size, while the original consistency threshold is set to 0.8. Logical condition combinations with sample size greater than or equal to 1 and original consistency greater than 0.8 can be retained. At the same time, the PRI consistency scores are re-encoded corresponding to a break observed in the distribution of consistency scores [77, 78]. When the PRI consistency is greater than or equal to 0.957, result variable 1 is retained. When the PRI consistency is less than 0.957, the result variable corresponding to the combination is manually changed to 0 [79]. Finally, complex solution, parsimonious solution, and intermediate solution are obtained, and the intermediate solution and parsimonious solution are nested for comparison. The final configuration results are shown in Table 7.

**Table 7:** Configuration combinations for the coordinated development between logistics and the economy.

Configuration	High coupling path			Non-high coupling path
	1	2	3	4
**II**	●	●	⊗	
**TI1**	●	●	●	⊗
**TI2**	●	●		⊗
**IS**	●		●	⊗
**HC**		⊗	●	⊗
**Consistency**	0.999	0.996	0.994	0.959
**Raw coverage**	0.665	0.362	0.438	0.853
**Unique coverage**	0.228	0.001	0.055	0.853
**Solution consistency**	0.996			0.959
**Solution coverage**	0.721			0.853

Notes: (●) indicate the presence of a condition, (⊗) indicate absence, blank spaces indicate “don’t care”. Large circles indicate core conditions; small ones, peripheral conditions.

### Configuration analysis

According to the results of the true table mentioned above, the consistency of the high-coupling path between regional logistics and economic development is 0.996. Table 7 reports three driving paths that represent possible conditional configurations, indicating that regions can promote highly coordinated development between logistics and the economy in all cases under the type of condition configurations. The overall coverage is 0.721, which is greater than 0.5, suggesting that the solution covers a substantial proportion of the variance in the region’s coordinated development between logistics and the economy. Moreover, the consistency of the non-high-coupling path between regional logistics and economic development is 0.959, indicating a high level of consistency. The overall coverage is 0.853, which is greater than 0.5, indicating a high level of explanatory power. Regardless of the high and non-high paths presented in the results, both the solution consistency and solution coverage values are above the threshold (0.75) [59], indicating the effectiveness of the empirical analysis. Therefore, the results of the configurational analysis in Table 7 can be used as a reference to explain the development coordination of regional logistics and the economy. Three high-coupling paths and one non-high-coupling path driving the synchronized progress of regional logistics and the economy can be identified accordingly.

As depicted in S3 Fig, a substantial disparity is evident in the condition variables namely: infrastructure investment (II), technological innovation of science and R & D (TI1), technological innovation of IT industry (TI2), industrial structure (IS), and human capital (HC) across different regions. The eastern region outperforms other regions in the first set of indicators, and the discrepancy widens as regions become more remote. These uneven developments pose challenges to the coordination between regional logistics and economic development. This finding aligns with previous research findings [9, 10].

### Robustness test

To ensure the robustness of the research findings, a robustness analysis will be conducted. Schneider and Wagemann [73] proposed three operations for conducting a robustness analysis: calibrating changes, modifying the consistency threshold, and adding or deleting cases. In this study, the fsQCA4.0 software was utilized to change the alternative calibration values of 0.75, 0.5, and 0.25. The results designated the same three configurations in the high coupling path with a slight alteration in consistency (0.989) and coverage (0.818). Similarly, two configurations in the non-high coupling path showed slight changes, with an increase in consistency (0.832) and coverage (0.952). The outcomes, as presented in Table 8, align essentially with those described in Table 7. These findings confirm the consistency between the conditional and original configuration outcomes, with no significant variations in consistency and coverage. Hence, the conditional configuration mentioned above can be deemed reliable, indicating the robustness of the study’s conclusions.

**Table 8:** The analysis result of the robustness test.

Configuration	High coupling path			Non-high coupling path
	1	2	3	4
**II**	●	●	⊗	
**TI1**	●	●	●	⊗
**TI2**	●	●		⊗
**IS**	●		●	⊗
**HC**		⊗	●	⊗
**Consistency**	0.993	0.972	0.984	0.952
**Raw coverage**	0.73	0.174	0.226	0.832
**Unique coverage**	0.514	0.003	0.085	0.32
**Solution consistency**	0.989			0.952
**Solution coverage**	0.818			0.832

Notes: (●) indicate the presence of a condition, (⊗) indicate absence, blank spaces indicate “don’t care”. Large circles indicate core conditions; small ones, peripheral conditions.

## Discussion

**Path 1** shows that high level of infrastructure investment (II), science and technology innovation (TI1), high-quality investment in technological innovation of the information technology industry (TI2), and industrial structure (IS) promote high harmonization of regional logistics and economy. Therefore, the type of this path is “infrastructure investment—technological innovation—industrial structure”, and the results are shown in S4 Fig.

According to the perspective of endogenous economic growth theory (new economic growth theory) [80], technological innovation is the source of economic growth, and the level of division of labor and accumulation of specialized human capital determine the level of technological innovation. The development of human capital has promoted technological innovation, particularly in the eastern region of China, gradually forming a development model driven by innovation. This can be explained by S3 Fig, which shows favorable indicators in terms of infrastructure investment, technological innovation, and industrial structure in the regions along this path. Under this path, regional logistics and the economy have achieved a high degree of coupling through a reliance on a favorable human capital environment, technological innovation levels, and sound infrastructure.

These cities are primarily concentrated in the eastern regions, just as shown in S3 Fig, particularly in the Yangtze River Delta and Pearl River Delta areas. For example, China’s major industrial agglomeration areas, including Guangdong, and Jiangsu, have established an undeniable dominance in industries such as communication equipment, computer and other electronic equipment manufacturing, and office machinery manufacturing. These regions heavily rely on other areas for their main energy and raw material requirements, resulting in a bustling flow of resources and products. Moreover, they have established well-regulated and standardized market environments, allowing for smooth element flows and efficient resource allocation. In terms of transportation, these regions are privileged with advanced airports, ports, and railway lines, giving them an unparalleled advantage over the Middle and Western regions of China. This is consistent with previous research [9, 81]. These regions have a large population and abundant human resources, advantageous geographical locations, convenient transportation, favorable locational advantages, sound infrastructure, and a solid industrial foundation. The government has placed high emphasis on their economic development. Industry clusters have already begun to form in these areas, supported by strong government policies. The concentrated development of these regions, coupled with their advantageous factors and government support, has led to seamless coordination between logistics and the economy, driving their sustained growth and prosperity. Consequently, the diversified development type facilitates a high level of coordination between logistics and the economy in these regions.

**Path 2** reveals that the coordinated development of regional logistics and economy can still be advanced on a broader scale, despite the presence of poor human capital, by emphasizing science and R & D (TI1), the information technology industry (TI2), and substantial infrastructure investment (II). This suggests that technological innovation and infrastructure investment hold a more significant influence as core factors in achieving a heightened level of coordination between regional logistics and the economy than other conditions. As a result, this path has been called “infrastructure investment—technological innovation type”, and the outcomes are illustrated in S5 Fig.

This finding further extends the view of Lan and Zhong [6]: Key factors like logistics infrastructure investment, and technological development level are the prerequisite for the coordinated development between logistics and the economy. One possible explanation is that the improvement of infrastructure investment leads to increased efficiency in necessary logistics-related activities. It enhances the process and effectiveness of the supply chain, resulting in reduced logistics costs, improved logistics service quality, and increased reliability. Put simply, the level of regional infrastructure development reflects the degree of logistics development to some extent. Moreover, infrastructure investment plays a critical role in fostering technological innovation by providing the necessary conditions for its development and implementation. Technological innovation raises the regional information level, introduces new technologies, and enhances efficiency in the region, further pushing the boundaries of economic growth and development.

The key regions in this path are Fujian and Liaoning. Fujian, as a coastal region, enjoys a favorable geographical advantage. According to the 2022 Report on Regional Science and Technology Innovation Evaluation in China, Fujian province ranks first alongside Guangdong and other 10 provinces and cities in terms of human resource input for scientific and technological activities. The number of research and development personnel in enterprises has risen to fifth place nationwide. This indicates the high level of importance placed on technological innovation in Fujian province, which plays a significant role in promoting regional coordinated development. According to the statistical yearbook of Fujian Province, the consistent emphasis in Fujian Province is on achieving high-level construction as an innovative province, focusing on bridging gaps in the field of scientific and technological innovation and implementing actions to enhance research and development investment throughout the society. It aims to accelerate the development of the tertiary industry and build a modern service sector, thereby stimulating greater vitality for high-quality development. Efforts are being made to deepen and promote multi-modal transportation integration through a unified system, accelerate the construction of the Fujian and Xiamen hubs, and create a trade hub for cross-strait trade in the Taiwan Strait.

Furthermore, as one of the economically strongest provinces in the Northeast region as shown in S3 Fig, Liaoning possesses favorable educational resources, a conducive human capital environment, and a foundation in the information industry. Based on the data presented in S3 Fig, it is evident that Liaoning Province has demonstrated a commendable performance across all indicators. In terms of the information industry and big data, Liaoning province ranks 12th nationwide and tops the list in the Northeast region. It emphasizes the cultivation of educational talents and technological innovation. As an exemplar of this path characterized by its emphasis on technological innovation and human capital, Liaoning province showcases its capacity for achieving significant synergy between regional logistics and economic development.

Indeed, in recent years, Liaoning Province has made remarkable advancements toward establishing itself as a hub for advanced industries and technological innovation. The province’s favorable geographical location and robust transportation network have enticed many enterprises to establish their operations in Liaoning, thereby bolstering the province’s economic growth. Furthermore, Liaoning Province has made substantial investments in research and development, particularly in cutting-edge sectors like aerospace, advanced equipment manufacturing, and biopharmaceuticals. These investments have not only propelled innovation but have also generated new employment opportunities and reinforced Liaoning’s pool of skilled human capital. The province’s unwavering focus on promoting technological innovation, nurturing a highly educated workforce, and harnessing the benefits of its strategic geographic location have been instrumental in catapulting it to become one of the leading economies in the Northeast region. It can be asserted that Liaoning Province has effectively fostered the coordinated development of the regional economy and logistics within this development model.

**Path 3** shows that even under the condition of poor infrastructure investment (II), a high level of science and R & D (TI1), industrial structure (IS), and human capital (HC) can still promote high coordination between regional logistics and economy. In other words, under this path, each region realizes the coordinated development of regional logistics and economy driven by multiple aspects of industrial structure, technological innovation, and human capital. Therefore, this path is called “technological innovation—industrial structure—human capital type”, and the main cities under this path are Shanghai and Hebei, as shown in S6 Fig.

In today’s rapidly evolving technological landscape, Hebei province has emerged as a frontrunner in industrial transformation by capitalizing on advancements in key sectors. The region has demonstrated significant progress in renewable energy, advanced manufacturing, and digitalization, paving the way for a robust and sustainable economic ecosystem. Through the application of emerging technologies to upgrade traditional industries, Hebei has successfully bolstered production efficiency, reduced costs, and improved supply chain coordination. As indicated in S3 Fig, Hebei’s industrial structure index reflects commendable performance in this regard, reinforcing its appeal as a destination for businesses seeking operational optimization. Furthermore, Hebei’s strategic investment in technological innovation has yielded tangible environmental benefits, as evidenced by its concerted efforts to develop clean energy alternatives and decrease reliance on fossil fuels, resulting in a reduced carbon footprint and the promotion of sustainable economic growth [82].

Moreover, Hebei’s pursuit of technological advancements and industrial transformation is closely intertwined with the overarching framework of the Beijing-Tianjin-Hebei integration initiative. S3 Fig demonstrates Hebei’s favorable score in the technological innovation index, highlighting its commitment to innovation. This initiative aims to foster collaboration and resource sharing among Beijing, Tianjin, and Hebei, thereby creating a closely connected and synergistic economic region. Notably, the development of advanced transportation and logistical infrastructure plays a pivotal role in this integration. Extensive efforts have been made to enhance connectivity and streamline the movement of goods, services, and people within the region. The implementation of innovative solutions such as intelligent transportation systems, smart logistics hubs, and multimodal transport networks has effectively optimized efficiency and mitigated bottlenecks in the supply chain.

As shown in S3 Fig, Shanghai’s indicators in the information technology industry (TI2) are quite high, and it also either scores well in industrial structure (IS), science and R & D (TI1), and human capital (HC). As China’s largest financial center, Shanghai is highly modernized. Its favorable economic environment provides great potential for the development of modern logistics. The development of logistics technology enables Shanghai’s logistics industry, as well as other logistics industries along the Yangtze River Delta, to develop in a healthy and balanced manner. At present, Shanghai has established a comprehensive transportation system consisting of various modes of transportation such as railroads, highways, and airways [54]. As a result, Shanghai’s investment index in infrastructure is significantly lower than that of other developed regions, but it is still among the ranks of primary coordination (0.694).

**Path 4**. In regions where regional logistics and the economy exhibit non-high coupling and coordination, the primary concentration is observed in western China and other remote areas. Despite noticeable improvements in economic performance in western regions in recent years, a significant developmental gap persists when compared to the eastern regions [9]. These areas face challenges such as inadequate infrastructure, technological innovation barriers, and substantial obstacles in talent development and recruitment (as shown in S3 Fig). Logistics plays a pivotal role in economic development and is of paramount importance in reducing economic disparities. Most western regions, situated inland, lack well-established infrastructure like highways, railways, and aviation, which severely impedes regional development. The lower educational levels in these regions hinder the cultivation of specialized talents and the establishment of innovative industries, resulting in the limited formation of regional industrial clusters. These factors, directly and indirectly, impede regional economic development and hinder the synchronized progress of the regional economy and logistics.

Historically, the fundamental industrial distribution orientation has revolved around manufacturing in East China and raw material production in West China. As illustrated in S7 Fig, regions within this trajectory exhibit a weak economic foundation. Among them, Tibet stands out due to its incomplete industrial system and a scarcity of manufacturing infrastructure. To mitigate these disparities and promote the coordinated development of regional logistics and the economy, it is imperative to reconfigure the layout of the manufacturing industry. This would involve redirecting resource processing and labor-intensive industries towards West China [81]. Such measures will help stimulate economic growth, promote job creation, and enhance the overall competitiveness of the western regions. Furthermore, strengthening regional cooperation and collaboration with the eastern regions is essential. This can be achieved through industrial coordination, resource complementarity, and market sharing. By fostering a supportive environment for business, innovation, and investment, it will facilitate the integration of regional logistics networks, bolster economic exchanges, and accelerate the growth of both regions.

In conclusion, while there have been improvements in economic performance in western and northeastern regions, there is a substantial need for further progress in achieving coordinated development between regional logistics and the economy. Overcoming challenges related to infrastructure, technological innovation, talent development, and industrial structure will be crucial in narrowing the economic disparity and fostering sustainable economic growth. By reconfiguring the manufacturing industry layout and promoting regional collaboration, it is possible to unlock the growth potential of these regions and achieve a more balanced and integrated development.

## Conclusions

This empirical analysis proposes an integrated framework based on fsQCA to identify the configuration of conditions that lead to the coordinated development between regional logistics and the economy. Initially, data pertaining to logistics and economic indicators across 31 provinces and cities are gathered and standardized to derive corresponding weights. Subsequently, the levels of logistics and economic development are assessed based on these weights. The coupling coordination degrees of each province and city are then computed to identify the variables. The observed high degree of coupling coordination in the eastern regions and the lower degree in the western and remote areas aligns with findings from prior studies [9]. These coupling coordination degrees serve as the outcome variable and undergo fuzzy set qualitative comparative analysis (fsQCA). Through this analysis, three paths of highly coordinated development, as well as one path representing non-highly coordinated development, are identified. Ultimately, the findings are comprehensively scrutinized and deliberated upon. The findings confirmed the four research propositions of this study.

First, compared to existing research, we not only analyze the unequal development of regional logistics and the economy among different provinces and cities in China but also treat the coupling coordination degree as the dependent variable to analyze the different causes of variations. This approach helps to intuitively identify the combinations of factors leading to different outcomes, enriching the theory of coordinated development between regional logistics and the economy. Second, the findings indicate that the coordinated development between regional logistics and the economy does not solely depend on a single condition. Instead, all three highly coordinated paths are influenced by multiple factors. This approach differs from traditional regression analysis, as it adopts a holistic and systemic perspective. The utilization of fuzzy set qualitative comparative analysis (QCA) in regional coordination development studies is enhanced, allowing for a more comprehensive understanding of the complex interplay between various factors. Third, the results show that there are three highly coordinated paths and one non-highly path. Specifically, to achieve a highly coordinated level between regional logistics and the economy, regions could adopt one of the following configurations: 1)“Infrastructure Investment—Technological Innovation”, 2) “Technological Innovation—Industrial Structure—Human Capital”, and 3) “Infrastructure Investment—Fundamental Innovation—Industrial Structure”. It can be obviously found that technological innovation, especially science and R&D (TI1) plays an important role in the region’s logistics and economy coordination, because of its existence as the core condition in all three highly coordinated paths, which is in line with the prior study [67, 83]. Technological innovation plays a crucial role in driving regional coordinated development in China. The spillover effects of technological advancements can be effectively harnessed and applied within well-established industrial systems. To this end, China has implemented a range of strategies, particularly in the eastern region of the country. For example, it actively pursued a shift from being known for “Made in China” to promoting a culture of “Created in China.”

The findings serve as a valuable reference for policy formulation and resource allocation in less developed regions, facilitating coordinated development between logistics and the economy.

### Policy recommendations

(1) Promoting differential technological innovation strategies
  Based on our research findings, it is evident that technological innovation plays a pivotal role in facilitating the coordinated development of regional logistics and the economy. Its significance cannot be underestimated. This study not only confirms but also expands upon prior research conducted by Zhou et al. [83]. In the case of China, the eastern region stands out due to its abundant human resources and technological advantages, which favor the integration of technology into its economy. Additionally, the availability of a large labor force and extensive markets allows the central region to adopt technology from the eastern region. Conversely, the western region lags behind in leveraging technological progress, mainly due to its lower level of economic development and a relative lack of adequate capital, human capital, and infrastructure. Given the disparities in resource endowments, economic infrastructure, and technological foundations among different regions, it is crucial for each region to identify and leverage their unique technological advantages. By prioritizing biased technological progress, less-developed regions can catch up with their more advanced counterparts, promoting overall coordinated development.
(2) Promoting talent cultivation and attraction
  The innovation-driven eastern region needs to strengthen the support and guidance of science and technology innovation policies, encourage enterprises to increase their R & D investment, and promote technological progress and the transformation of innovative achievements. At the same time, talent training and attraction should be increased to attract high-quality talents to flow into innovative industries and enhance regional innovation capacity and competitiveness. In the resource-based western region, the government can take measures to encourage resource flows and regional cooperation. For example, it should promote cross-regional resource allocation and circulation, and encourage cooperation and synergistic development among enterprises. At the same time, it should strengthen infrastructure construction, improve transportation and logistics efficiency, and provide convenient conditions for resource development and circulation.
(3) Promoting rational allocation of resources
  It is apparent that different regions with varied resource endowments should have comparative advantages, it is crucial to ensure that the conditions for development in priority regions are met when allocating resources. This means that resources can be appropriately transferred to relatively less developed areas while meeting the development needs of these priority regions. By doing so, it is possible to accelerate the development of infrastructure in underdeveloped regions and narrow the development gap between different regions.
(4) Optimize industrial structure
  The government should also focus on the impact of industrial structure on regional development between logistics and the economy. It should promote the optimization and upgrading of the economic structure through the formulation of targeted industrial policies, so as to form a pattern of complementary and synergistic development between resource-based and innovation-driven regions. Governments and policy researchers can promote the balanced development of the economies of different regions by regulating resource allocation and promoting interregional industrial cooperation.

### Limitations and future research

This study focused on the path analysis of the coordination development levels between regional logistics and the economy in 31 provinces and regions across China. Although it has valuable contributions, the study does possess certain limitations. This paper analyzes the data from the latest year using a static perspective. In future research, a multi-year, long-term approach can be adopted, which not only enables us to obtain a static regional difference level but also reveals the development change curve of each region over a long period of time. This is conducive to promoting the coordinated development of logistics and economy in different regions, and to formulating multidimensional plans to drive the development of each region, reduce regional differences, and promote the integrated development between cities and cities, regions, and regions. Consequently, it is recommended that future investigations incorporate data from multiple years to facilitate a more thorough and in-depth analysis.

## Supporting information

S1 Fig Research framework. (TIF)

S2 Fig Further analysis of the potential necessary condition. (TIF)

S3 Fig Stacked chart of various conditional variables. Divided into four parts based on the East, Center, West, and Northeast regions.
(TIF)

S4 Fig Path 1. The dots in the figure represent different provinces: 0)Guangdong,1)Zhejiang,2)Jiangsu,3)Shandong,4)Anhui,5)Beijing,6)Fujian,7)Hubei,8)Henan,9)Hunan,10)Sichuan.
(TIF)

S5 Fig Path 2. The dots in the figure represent different provinces: 0)Fujian, 1)Liaoning.
(TIF)

S6 Fig Path 3. The dots in the figure represent different provinces: 0)Hebei, 1)Jiangxi,2)Shanghai.
(TIF)

S7 Fig Path 4. The dots in the figure represent different provinces: 0)Tibet, 1)Qinghai, 2)Ningxia, 3)Hainan, 4)Jilin, 5)Gansu, 6)Shanxi, 7)Heilongjiang.
(TIF)

S1 File The data of conditional variables. (XLSX)

S2 File The data of outcome variables. (XLSX)
