# Supplementary figures and images for: Path analysis of regional logistics and economy coordinated development: An fsQCA approach
Source: PLoS One. 2024 Feb 1;19(2):e0297441. doi: 10.1371/journal.pone.0297441 (PMC10833549; doi:10.1371/journal.pone.0297441)

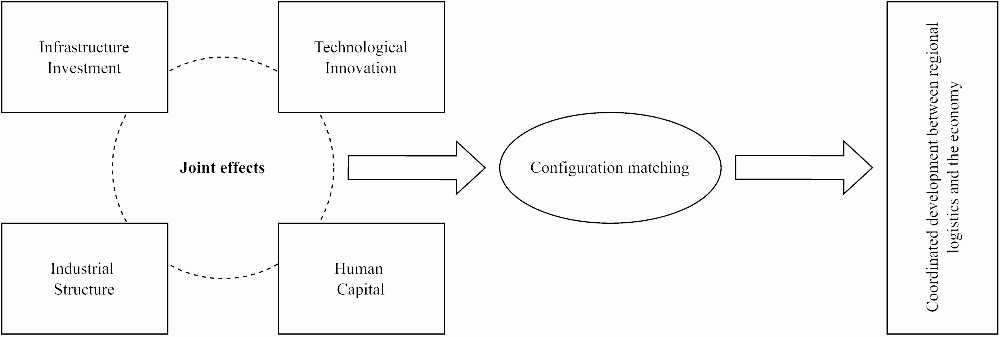

Supplement: S1 Fig — (TIF) [file pone.0297441.s001.tif]
